# Editorial: How Plants Deal with Stress: Exploration through Proteome Investigation

**DOI:** 10.3389/fpls.2017.01176

**Published:** 2017-06-30

**Authors:** Dipanjana Ghosh, Qingsong Lin, Jian Xu, Hanjo A. Hellmann

**Affiliations:** ^1^School of Pharmacy and Research, People's UniversityBhopal, India; ^2^Department of Biological Sciences, National University of SingaporeSingapore, Singapore; ^3^School of Biological Sciences, Washington State UniversityPullman, WA, United States

**Keywords:** proteomics, abiotic stress, biotic stress, plant stress biology, heavy metal stress

Biotic and abiotic stress factors serve as consistent threats to a plant's life cycle. Biotic stress factors such as damage through pathogens or herbivore attack as well as abiotic stress factors like variation in temperature, rainfall, and salinity, have placed the members of the plant kingdom under constant challenges for their survival. As a consequence, global agricultural and horticultural productivity has always been below its optimal capacity.

Being sessile in nature, plants cannot escape from the stress, instead they acclimatize to adverse condition by adopting certain systemic changes. These changes include developmental and physiological alterations which influence the genome, proteome, and metabolome of the plant. Since proteins are key regulators of cellular responses, investigations into proteome alterations during stress induction as well as recovery, can provide important information on how plants cope with stress factors. The process has been widely discussed in this Research Topic which comprises nineteen research articles, two reviews, one mini review, and one opinion article.

A significant portion of the research articles included in this Research Topic investigated proteome level alterations in diverse organ parts of the plant body upon exposure to abiotic stress factors such as drought, salinity, and submergence. Comparative proteomic investigations between drought and submergence stress showed that bermudagrass adopt a quiescence strategy in a prolonged submerged state by declining metabolic activities, whereas in a drought environment plants increase their tolerance level through higher levels of photosynthesis and redox potential (Ye et al.). Enhanced levels of molecular chaperones were found to be associated with drought tolerance in multiple studies (Ye et al.; Zhao et al.; Chmielewska et al.) performed on different plant species (bermudagrass, corn, and barley, respectively). Interestingly, when maize plants were challenged by the combination of both drought and heat, up-regulation of a specific set of ethylene-responsive, and ABA-, stress-, and ripening-inducible-like proteins was observed. However, individual exposure to drought or heat stress did not show the above proteome alterations, indicating that these proteins are specifically required to resist the combinatorial effects of the above two stresses (Zhao et al.). Consistently, over-expression of glycolate oxidase (GLO) was found during adaptation of rice plants to both high-light intensity and high temperature but not to high-light intensity alone, with H_2_O_2_ and salicylic acid being the signaling molecules that mediate the adaptive responses (Cui et al.). Studies on salt stress reported the up-regulation of mainly defense-related proteins and proteins related to enhanced energy metabolism (Maršálová et al.; Long et al.). In addition, a quantitative proteomics study explored that vacuolar ATPase AVP1 and sugar transporters of the ERDL (early responsive to dehydration-like) family and TMT2 (tonoplast monosaccharide transporter 2) were upregulated salt stress treatment (Pertl-Obermeyer et al.). Notably, two plant growth-promoting bacteria signal compounds, lipo-chitooligosaccharide (LCO), and thuricin17 (Th17) were found to have a stronger effect on proteins related to the carbon and energy metabolic pathways in salt-treated plants when compared to mock-treated Arabidopsis plants (Subramanian et al.). These compounds thus hold promise for the development of potent agrochemicals that could improve crop productivity under salt stress.

Comparison of growth rate and stress responses between wild type and forest grown varieties of the same plant were investigated through proteomics investigations on Ginseng (Ma et al.) that revealed protein profiles of 25-year-old forest Ginseng is comparable to that of younger wild Ginseng and differentially expressed proteins were involved in energy metabolism, ginsenoside biosynthesis, and stress response.

Heavy metal induced stress is another critical challenge for agricultural productivity. Proteomic investigations on heavy metal induced rice varieties derived that phospholipase D activity is positively correlated with cadmium stress and Cu-binding proteins might help to ameliorate the effects of copper stress. In addition, a wide range of differentially expressed protein datasets have been reported for plants exposed under zinc and nitric oxide (NO) stress (Yang et al.; Wang et al.; Wang et al.; Zhang et al.).

Biotic stress such as fungal infection also contributes toward proteomic changes. Resistance against those fungal pathogens was found to be correlated with the abundance of amylase inhibitor proteins, leucine-rich repeats, and cysteine-containing secreted small proteins found in xylem sap as derived in proteomics investigations on cabbage, tomato, and Triticale plants challenged by several fungal species of the Fusarium genus (Perlikowski et al.; Pu et al.; Gawehns et al.).

Other than the above-mentioned research highlights, Opinion and review articles included in this Research Topic covered various important aspects related to plant stress responses at the protein level. These include vascular sap proteomics for long-distance signaling (Carella et al.), hydrogen sulfide as a signaling molecule in plant cross adaptation (Li et al.), the importance of proteomics in understanding crop stress tolerance (Ahmad et al.), and increasing confidence of proteomics data for identifying stress-responsive proteins in crop plants (Wu et al.; Wu and Wang).

Overall, this Research Topic has provided a wealth of information in the field of plant stress biology from a proteomics perspective. Functional characterization of some of the candidate proteins and pathways may ultimately contribute to the development of novel breeding strategies that will improve agricultural productivity in a changing climate.

## Author contributions

DG: Contribute in compilation of concepts from individual articles of the research topic, manuscript write up and revision; QL and JX: Contributed to the revision of the manuscript write-up; HH: Provided guidance during manuscript write up and contributed to the revision of the manuscript draft.

### Conflict of interest statement

The authors declare that the research was conducted in the absence of any commercial or financial relationships that could be construed as a potential conflict of interest.

